# Ivermectin Identified Using a High-Throughput Screening System Exhibits Anti-*Clonorchis sinensis* Activity in Rats

**DOI:** 10.3390/antibiotics14080837

**Published:** 2025-08-19

**Authors:** Soon-Ok Lee, Hyeryon Lee, Ki Back Chu, Jianhua Li, Sung-Jong Hong, Sung Soo Kim, Joo Hwan No, Fu-Shi Quan

**Affiliations:** 1 Department of Medical Zoology, School of Medicine, Kyung Hee University, Seoul 02447, Republic of Korea; 2 Medical Research Center for Bioreaction to Reactive Oxygen Species and Biomedical Science Institute, School of Medicine, Graduate School, Kyung Hee University, Seoul 02447, Republic of Korea; 3 Chemical and Structural Biology of Pathogens, Institut Pasteur Korea, Seongnam 13488, Republic of Korea; 4 Department of Parasitology, College of Medicine, Inje University, Busan 47392, Republic of Korea; 5 Department of Infectious Disease and Malaria, Paik Institute of Clinical Research, Inje University, Busan 47392, Republic of Korea; 6 State Key Laboratory for Diagnosis and Treatment of Severe Zoonotic Infectious Diseases, Key Laboratory for Zoonosis Research of the Ministry of Education, Institute of Zoonosis, College of Veterinary Medicine, Jilin University, Changchun 130062, China; 7 Department of Medical Sciences, College of Medicine, Chung-Ang University, Seoul 06974, Republic of Korea

**Keywords:** *Clonorchis sinensis*, high-throughput screening, ivermectin, rat

## Abstract

**Background:** Clonorchiasis, caused by the parasite *Clonorchis sinensis*, remains a public health concern in East Asian countries. **Methods:** In this study, high-throughput screening was used to analyze 320 compounds for potential inhibitory activity against *Clonorchis sinensis*. To ensure the selection of high-confidence hits, a stringent inhibition threshold of 80% was applied, leading to the identification of three active compounds: moxifloxacin, hexachlorophene, and ivermectin (IVM). Ivermectin emerged as a hit compound and was assessed for its anti-*C. sinensis* efficacy. **Results:** Ivermectin demonstrated dose-dependent trematocidal activity against *C. sinensis* metacercariae (CsMC) and newly excysted juveniles (CsNEJs), showing superior efficacy against CsMC and CsNEJs compared to praziquantel. To assess in vivo efficacy, rats were infected with CsMC and treated with ivermectin at 1 and 4 weeks post-infection (wpi) to target larval and adult stages, respectively. A significant worm burden reduction was observed compared to untreated control when treatment was administered at 1 wpi, showing an antiparasitic effect against larvae. Parasite-specific IgG levels and ALT/AST responses were comparable to those of the infection control group. **Conclusions:** These findings suggest that ivermectin may serve as a potential alternative drug targeting *C. sinensis* larvae.

## 1. Introduction

Clonorchiasis, caused by the parasitic flatworm *Clonorchis sinensis*, is a major food-borne zoonosis. Currently, more than 200 million individuals are at risk with 15 million cases reported globally [1,2,3]. The disease is endemic in several Asian countries, particularly in East Asia, including China, Korea, Taiwan, and northern Vietnam. In Korea, clonorchiasis persists in major river basins such as Nakdong, Seomjin, Geum, Yeongsan, and Han rivers [4,5]. *C. sinensis* is associated with various biliary diseases, most notably cholangiocarcinoma (CCA), and is classified as a Group 1 carcinogen by the International Agency for Research on Cancer [6]. The strong epidemiological link between *C. sinensis* and CCA, supported by several case–control studies, underscores its public health impact [7,8,9]. Current control strategies primarily rely on mass drug administration of the broad-spectrum anthelminthic praziquantel (PZQ) [10,11], which has been shown to be generally safe and effective when administered as a single oral dose, as supported by numerous studies [12,13]. Nonetheless, the reliance on this single therapeutic agent raises significant concerns regarding the potential emergence of drug resistance [14,15]. Moreover, praziquantel (PZQ) is effective for adult stages of all medically important liver flukes, but relatively ineffective for larval stages in vivo and in vitro [16,17]. This means that treatment does not eliminate all parasites from infected patients, and repeated treatment is required. These challenges underscore the urgent need for novel therapeutic strategies to improve treatment outcomes, enable sustained disease control and ultimately achieve eradication.

The majority of existing anthelmintic compounds have been identified using in vivo screening in animal models, which is a labor-intensive, low-throughput, and often subjective process that slows the discovery of novel compounds [18,19]. Consequently, there is a need for new trematocidal agents that are active against all life stages, which has led to renewed interest in drug discovery research using both phenotypic and target-based approaches. This includes the development and application of whole-organism screens for compound testing [20], target-based drug discovery [21], and identification of putative molecular targets through annotated trematode sequence analysis [22]. Among them, target-based high-throughput screening (HTS) has attracted considerable interest, and its ap-plication in identifying novel antischistosomal drug candidates has been demonstrated [21,23].

While the minimal compound requirement is a favorable feature of this approach, its broader application is limited by incomplete knowledge of parasite biology, challenges in target protein expression, and high associated costs. Hence, recent drug discovery efforts have increasingly focused on HTS of larval-stage parasites [24,25]. In support of this notion, several studies reported the use of artificially derived larval-stage worms and newly transformed schistosomula (NTS) from *S. mansoni* for antischistosomal drug discovery [26,27]. These research models have enabled early-stage compound evaluation and con-tributed to identifying potential candidates from large-scale compound libraries. Supported by image-based automated microscopy, HTS involving these research models has enabled early-stage compound evaluation and facilitated the identification of candidates that affect parasite motility and morphology in large-scale compound libraries [28,29]. However, HTS-based anthelmintic development using Schistosoma spp. is limited, as targeting larval stages that parasitize in aquatic environments does not accurately reflect the biological characteristics of *C. sinensis* and other trematodes. To address this, we performed HTS on a compound library to identify potential therapeutic hits with anti-parasitic activity against the excysted metacercariae stage of *C. sinensis*. Here, we report for the first time that ivermectin (IVM) exhibits significant in vitro and in vivo activity among the identified hits.

## 2. Results

**Figure 1:**
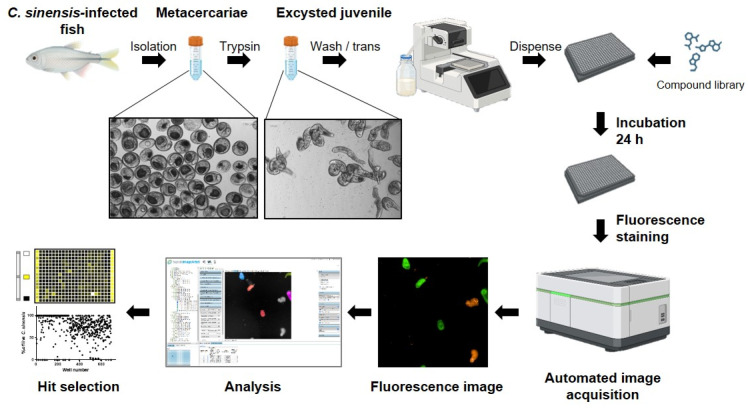
Schematic overview of in vitro screening and in vivo assay for the discovery of anti-*C. sinensis* compounds. Live metacercariae were isolated from infected freshwater fish. CsMC were excysted using trypsin, and most of the juveniles were released. CsNEJs were dispensed into the 384-well plate containing the test compounds. The parasites were stained with fluorescent dye 24 h after compound treatment and imaged immediately. Fluorescence images were acquired using an automated high-throughput screening system, and morphological features were extracted from multi-channel fluorescence microscopy images. Hit compounds were selected based on the percentage of live *C. sinensis* per well, calculated from the analyzed data. After confirmatory dose-response testing in vitro, an efficacy test was performed in a rodent model.

**Figure 2:**
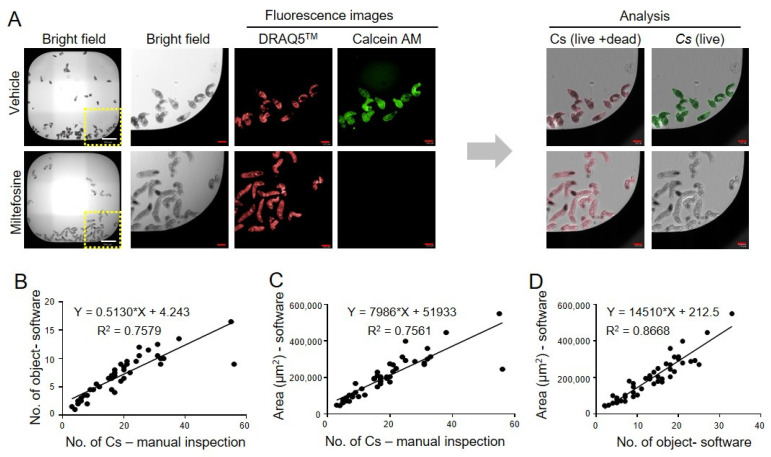
Analysis of live/dead ratio for anti-*C. sinensis* compound discovery assay. (**A**) Image acquisition and analysis of *C. sinensis* stained with calcein AM and DRAQ5. Calcein AM stains only live organisms with esterase activity, while DRAQ5 stains all the parasites. The white scale bar indicates 500 μm, and the red scale bar indicates 100 μm. The parasites in the yellow box on the left were enlarged and seen on the right bright field. (**B**,**C**) The quantification method was validated by comparing the DRAQ5-stained area (or the number of objects) obtained with software analysis and the number of *C. sinensis* counted by manual inspection. (**D**) The DRAQ5-stained area was increased in proportion to the number of *C. sinensis*.

*Establishment of a HTS system for CsNEJs.* An inhibitor screening system was established using CsNEJs in a 384-well plate (Figure 1). As in vitro cultivation of *C. sinensis* is not feasible, CsMC were isolated from wild-caught fish. Following trypsin exposure, NEJs were obtained and dispensed into 384-well plates. Parasites were stained with fluorescent dyes and analyzed using automated imaging. Both live and dead parasites were stained with DRAQ5 DNA stain, while live parasites were selectively stained with calcein AM. CsNEJs treated with miltefosine served as the positive control group, in which calcein AM fluorescence was not observed. In contrast, DMSO-treated negative controls exhibited strong calcein AM signals overlapping with DRAQ5. These results validated the ability of the assay to reliably distinguish live parasites from dead ones (Figure 2A).

Next, we assessed the accuracy of automated parasite detection through quantitative image analysis. The correlation between the image-detected and manually counted parasite numbers had an R^2^ value of 0.55. The image-detected parasite count was approximately 0.5-fold lower than the manual count, as reflected by a regression slope of 0.5130. For parasite area measurements, the correlation with the manual count was slightly stronger (R^2^ = 0.66). Notably, error bars were smaller for parasite area quantification than for parasite counts, indicating greater consistency in area-based detection.

**Figure 3:**
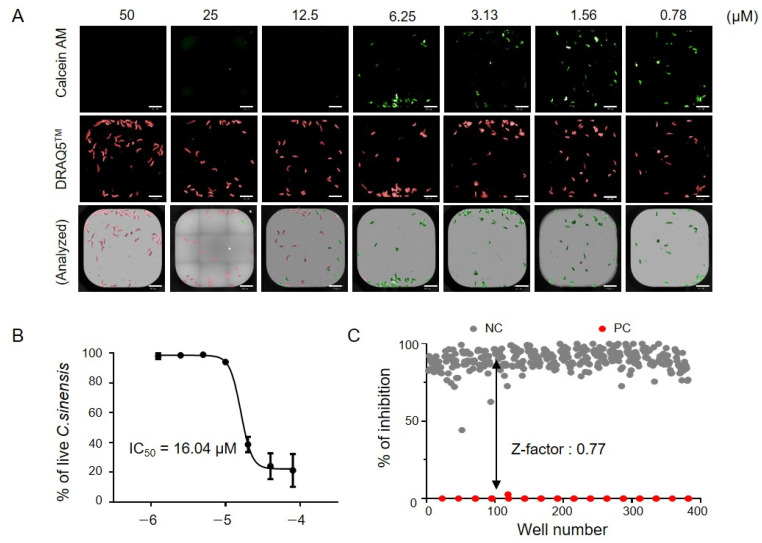
Quality control and negative control for the screening. (**A**) Representative im ages of *C. sinensis* after miltefosine treatment. Miltefosine showed an immediate anti-*C. sinensis* effect. Red indicates the DRAQ5-stained area and green indicates the calcein AM-stained region. The scale bar indicates 500 μm. (**B**) Dose-response curve of miltefosine. (**C**) The % inhibition of negative and positive wells is shown. The Z′ factor of the representative experiment was 0.77 in the screening system.

*Validation of HTS-based CsNEJs inhibition assay.* The HTS system and quantitative image analysis were validated using a serial dilution of miltefosine. CsNEJs, regardless of their viability, were effectively stained with the DNA dye DRAQ5 at all tested miltefosine concentrations, while viable parasites stained with calcein AM fluorescence were only observed at lower concentrations (Figure 3A). For quantitative analysis, calcein AM-positive areas representing viable parasites were normalized to the total DRAQ5-stained area, which included both live and dead parasites, to minimize well-to-well variability without relying on independent positive and negative control wells.

Based on image-derived measurements, the half-maximal inhibitory concentration (IC50) of miltefosine was determined to be 16.4 µM (Figure 3B). Further evaluation of the system in a full 384-well plate format yielded a Z′ factor of 0.77 and a coefficient of variation of 7.10 for the DMSO-treated control wells, confirming the robustness and reproducibility of the assay.

**Figure 4:**
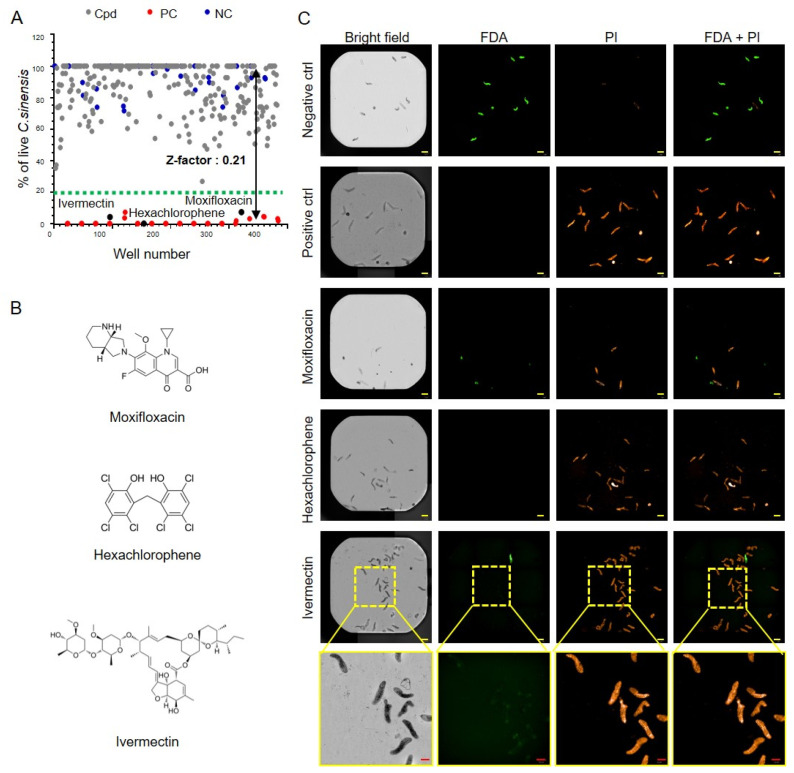
Pilot screening of compound library. (**A**) Overview of the primary screen. (**B**) Compound structures with >80% inhibition. Representative images of negative (vehicle) and positive (curcumin) controls. The yellow scale bar indicates 200 μm, and the red scale bar indicates 100 μm. (**C**) Representative images of *C. sinensis* 24 h after compound treatment. Selection criteria include not only the percentage of inhibition but also visual inspection, toxicity, and pharmacokinetic properties of the compounds. IVM was selected for further evaluation in dose–response and time-dependent assay.

*HTS of a small molecule library against CsNEJs*. A pilot screening of 320 compounds was performed to assess trematocidal activity against CsNEJs. For this screen, DRAQ5 was replaced with PI to reduce the washing step, and FDA was used in place of calcein AM due to its lower sensitivity to paraformaldehyde fixation. The Z′ factor for this screen was 0.21, which was lower than that of the validation assay. Therefore, a stringent threshold of 80% inhibition was applied that resulted in the selection of three hit compounds: moxifloxacin (92.77% inhibition), hexachlorophene (100%), and ivermectin (95.88%) (Figure 4A). Chemical structures for the three hit compounds are also provided (Figure 4B). Automated imaging showed a clear absence of FDA fluorescence in compound-treated wells, while PI-stained parasites were detected (Figure 4C). As expected, PI-stained parasites were not observed in the DMSO-treated negative control group. Given its clinical relevance as an antiparasitic agent, ivermectin was selected for further confirmation and analysis.

**Figure 5:**
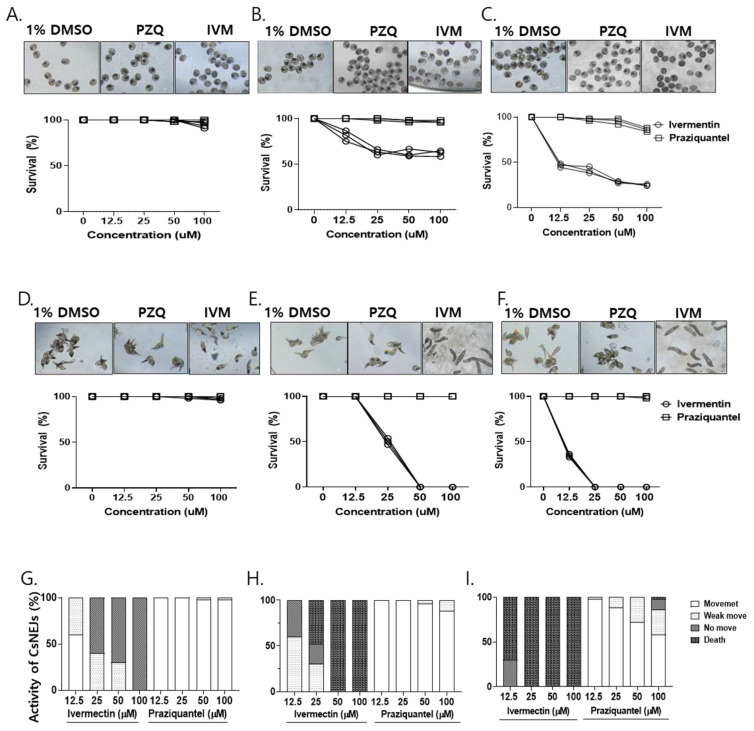
Assessing the larvicidal effect of ivermectin against CsMC and CsNEJs in vitro. Metacercariae and excysted larvae were treated with 12.5, 25, 50, and 100 µM concentrations of IVM or PZQ. Anti-metacercarial activity of the drugs was determined at 24, 48, and 72 h post-drug treatment for different doses of ivermectin (**A**–**C**). The larvicidal effects of ivermectin against CsNEJs were observed after 6, 12, and 24 h of drug exposure (**D**–**F**). To observe the effects of IVM and PZQ on the motility of CsNEJs, the juvenile worms were exposed to drug concentrations of 12.5, 25, 50, and 100 µM for 24 h and categorized into four stages: dead, viable but immobile, weak movement, and vigorous movement (**G**–**I**).

*IVM showed anti-metacercarial and anti-CsNEJs effects.* Trematocidal efficacy of ivermectin against CsMC and CsNEJs was assessed using optical microscopy. CsMC were exposed to ivermectin at concentrations of 12.5, 25, 50, and 100 μM for 24, 48, and 72 h. No trematocidal effect was observed at any of the tested concentrations after 24 h. Upon 48-h exposure, ivermectin exhibited dose-dependent efficacies of 0%, 36.9%, 38.2%, and 38%, respectively. By 72 h, the corresponding efficacies for the four doses increased to 53.4%, 58.6%, 74.7%, and 70.1% (Figure 5A–C). In CsNEJs treated with ivermectin, no trematocidal effect was observed at any of the concentrations during the first 6 h. By 12 h, however, ivermectin induced 49.7% and 100% efficacy at 25 and 50 μM, respectively. After 24 h, 65.4% and 100% were observed at 12.5 and 25 μM, respectively (Figure 5D–F). Larval motility was assessed at 6, 12, and 24 h post-treatment. At 6 h, 60–100% of CsNEJs were immobile at concentrations of 25–100 µM, although no mortality was observed. After 12 h, 48–100% of the larvae were dead at these concentrations. At 24 h post-treatment, 70% mortality was observed at 12.5 µM, and all CsNEJs were dead at higher concentrations. In contrast, PZQ treatment at 100 µM resulted in a 12% reduction in larval motility after 24 h (Figure 5G–I).

**Figure 6:**
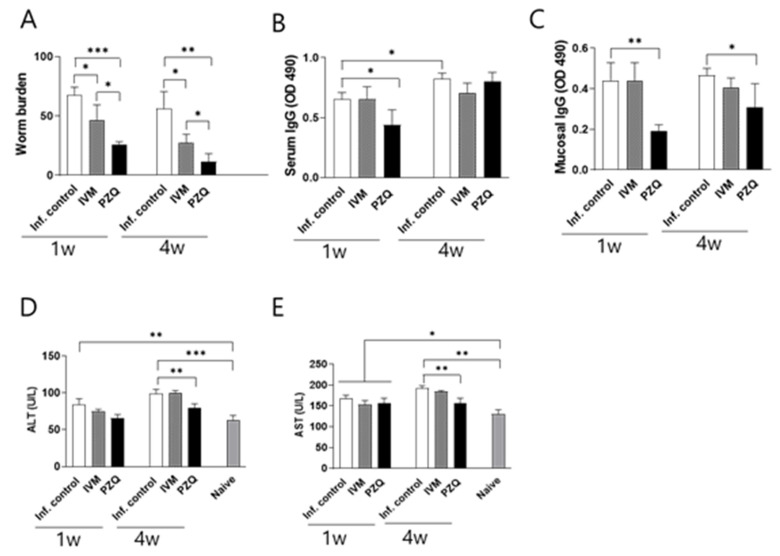
Worm burden, antibodies, and liver damage assessment. Samples were harvested following two different treatment time points. Adult *C. sinensis* worms were recovered from the bile ducts of rats and quantified to calculate worm recovery (**A**). ELISA was performed using sera (**B**) and duodenal samples (**C**) of rats to determine *C. sinensis*-specific antibody responses. ALT (**D**) and AST (**E**) concentrations in serum samples were evaluated. Statistical significance was indicated using asterisks (* *p* < 0.05, ** *p* < 0.01, *** *p* < 0.001).

*Ivermectin induced anti-C. sinensis efficacy in rats*. CsMC-infected rats were treated at 1 and 4 wpi and subsequently euthanized 4 weeks after treatment for worm recovery from the bile ducts. The worm recovery rate in the untreated control group was 67.5%. In contrast, at 1 wpi, the worm reduction rate was 53.5% in the IVM-treated group and 68.5% in the PZQ-treated group. By 4 wpi, the reduction rates increased to 63.5% and 88.5% for IVM and PZQ, respectively (Figure 6A). Parasite-specific IgG responses were detected in all groups. At 5 wpi, serum IgG levels in the PZQ-treated group were significantly lower than those in the infection control group (* *p* < 0.05), while those in the IVM-treated group were comparable to controls (Figure 6B). At 8 wpi, serum IgG levels were comparable across all groups. In contrast, mucosal IgG levels were significantly lower in the PZQ treatment group at both treatment points (* *p* < 0.05, ** *p* < 0.01) (Figure 6C), where lower worm burdens were detected. In the IVM-treated group, mucosal IgG levels did not differ significantly from those of the infection control group at any time point. Furthermore, no significant differences were observed between the two treatment groups. These results suggest that mucosal IgG responses correlate more closely with worm burden than serum IgG levels, indicating greater sensitivity of the mucosal response. Liver function was evaluated by measuring serum levels of ALT and AST, which are enzymes released into circulation during hepatocellular damage and commonly used as indicators of hepatobiliary disease. Baseline ALT levels increased from 62.74 U/L to 80.19 U/L and 106.6 U/L at 5 and 8 wpi with *C. sinensis*, respectively (* *p* < 0.05, *** *p* < 0.001). At 1 wpi, ALT levels in the treatment groups did not significantly differ from those in the infection control. By 5 wpi, a significant reduction was only observed in the PZQ-treated group, but not in the IVM-treated rats (*** *p* < 0.001). A similar pattern was observed for AST. AST levels in the naïve group were 130.1 U/L, increasing to 167.4 U/L by 5 wpi and remaining elevated through 8 wpi with *C. sinensis* (** *p* < 0.01, *** *p* < 0.001). AST levels in the treatment groups at 5 wpi did not significantly differ from those in the infection control, whereas significant reductions were observed in the PZQ-treated rats at 8 wpi (*** *p* < 0.001) (Figure 6E). These results suggest that treatment with 30 mg/kg IVM and 250 mg/kg PZQ does not induce hepatotoxicity and may help preserve liver function during clonorchiasis.

## 3. Discussion

Despite significant advancements in phenotypic screening for STH, similar approaches for *C. sinensis* have been limited. This limitation is primarily due to restricted access to parasite eggs, which cannot be propagated in large quantities under laboratory conditions, and the lack of well-established phenotypic screening models. To address these challenges, we adapted wild-caught CsMC for use in a 384-well plate format and employed fluorescent labeling combined with HTS imaging. Fluorescent staining enabled clear differentiation between live and dead larvae, and image analysis provided a quantitative assessment of parasite viability. The assay was validated using the reference compound miltefosine, yielding a Z′ factor of 0.77, which confirmed the robustness and reliability of the platform. The Z′ factor obtained in the pilot screen of 320 compounds was lower than that observed during the validation assay (Figure 2), likely due to reduced parasite viability during screening. This interpretation was supported by the increased variability observed among the negative control wells (Figure 3). Although the Z′ factor was reduced, it remained within an acceptable range for primary screening. To ensure the selection of high-confidence hits, a stringent inhibition threshold of 80% was applied, leading to the identification of three active compounds: moxifloxacin, hexachlorophene, and IVM. Among the screened compounds, hexachlorophene exhibited moderate activity in in vitro studies against *C. sinensis* [30]. Moxifloxacin, a fourth-generation synthetic fluoroquinolone antibiotic, is commercially available as an ophthalmic solution for the treatment of bacterial conjunctivitis. Recently, it has been shown to shorten the treatment duration of pulmonary tuberculosis when used in combination with rifapentine; however, its antiparasitic potential has not yet been elucidated [31].

IVM is effective against a variety of parasitic diseases such as lymphatic filariasis [32]. Its mechanism of action involves the activation of glutamate-gated chloride channels in parasites, leading to an influx of chloride ions, hyperpolarization of neuronal membranes, and subsequent disruption of neuromuscular transmission, resulting in parasite paralysis and death [33,34]. In addition to its established use, IVM has demonstrated therapeutic potential against other parasitic diseases such as malaria [35,36], trypanosomiasis [37], schistosomiasis [38], trichinosis [39], and leishmaniasis [40]. However, its efficacy against liver flukes has not yet been investigated. IVM was selected from these candidates for further evaluation based on its well-established use as a broad-spectrum anthelmintic and subsequently assessed in both in vitro and in vivo studies. In the present study, IVM exhibited potent trematocidal activity against CsNEJs. The anti-larval effect of IVM appears to be significantly greater against CsNEJs than CsMC. This difference may be attributed to the presence of a cyst wall encapsulating the larvae in CsMC. Moreover, since the structural characteristics of metacercarial cyst wall also vary among trematodes, such differences may influence anthelmintic susceptibility.

These findings are consistent with earlier findings, which demonstrated the molluscicidal activity of IVM against *Biomphalaria glabrata*. Specifically, a concentration of 0.2 μg/mL of IVM was sufficient to kill 50% of cercariae and miracidia within 5 min, and this mortality rate increased to approximately 90% after 30 min [38]. This effective dose is substantially lower than the 25 μM required to achieve a larvicidal effect against *C. sinensis*. In *Fasciola hepatica*, near complete ovicidal activity was observed following treatment with 2.0 mM IVM [41]. Although the mechanism underlying these interspecies differences remain unclear, they may be attributed to genetic and molecular factors influencing drug susceptibility. Finally, IVM-treated CsNEJs demonstrated progressive reductions in motility proceeding death, supporting its concentration- and time-dependent trematocidal effect (Figure 4). In in vivo experiments, IVM at a near-toxic dose (30.1 mg/kg) was orally administered to rats infected with *C. sinensis*. No immediate signs of toxicity were observed. In the group treated at 1 wpi, the worm reduction rate was 53.5%, indicating partial efficacy. However, a significant reduction in worm burden was not observed in rats treated at 4 wpi.

IVM is a macrocyclic lactone that exerts its anthelmintic effect primarily by targeting glutamate-gated chloride channels, which are expressed in arthropods, nematodes, and trematodes [42]. Although this drug has been approved for the treatment of filarial infections and is effective against nematodes and certain ectoparasites, its limited efficacy against cestodes and trematodes restricts its broader therapeutic application [43]. This reduced efficacy has been attributed to the absence or limited role of gamma-aminobutyric acid-mediated neurotransmission in these parasite groups. Nonetheless, IVM has been considered for the treatment of trematode infections in several studies [44,45]. The discovery of glutamate-mediated signaling pathways in *S. mansoni* has renewed interest in targeting glutamate-gated chloride channels in trematodes [46].

In our study, IVM exhibited a trematocidal effect when administered at 1 wpi, but not at 4 wpi. This stage-specific efficacy may be attributed to differential expressions of glutamate-gated chloride channels, target distribution, drug penetration, and metabolic processes between larval and adult stages of *C. sinensis*. Similar findings have been reported in *Trichobilharzia ocellata* and *S. mansoni* [47]. Following *C. sinensis* infection, serum IgG levels increased in all infected rats. Rats receiving PZQ at 1 wpi experienced a significant reduction in parasite-specific IgG, whereas no such reductions occurred in IVM-treated groups. At 4 wpi, neither treatment affected serum IgG levels. In contrast, mucosal IgG levels decreased in both 1- and 4-week PZQ groups but remained unchanged in the IVM groups. These results suggest that the mucosal immune response is a more sensitive marker of treatment response than serum IgG and that early intervention influences the host immune response, while late treatment has little immunomodulatory effect. This is consistent with findings from *S. mansoni* infection studies in mice, where IVM treatment did not alter IgG levels or granuloma formation [48]. Liver enzyme analysis further supported the infection timeline and treatment effects. ALT/AST levels, biomarkers of hepatocellular damage, were significantly higher at 4 wpi compared to 1 wpi. Both enzyme levels were significantly reduced in the 4-week PZQ treated rats compared to the infection control group. At 1 wpi, ALT/AST levels in both infection control and treatment groups remained significantly higher than those of the naïve, suggesting that liver damage occurs early in the infection and may be partially reversed through drug treatment.

Although IVM is generally considered ineffective against trematodes, several studies have explored its potential in combination therapies for trematode infections. Clinical trials have shown that single-dose IVM, when co-administered with other drugs, produced mild effects on *S. haematobium* but had no impact on *S. mansoni* [49,50]. More recently, IVM and diaryl dichalcogenides demonstrated trematocidal activity against *F. hepatica* [51]. These reports support the concept of using IVM in combination therapies to broaden the activity spectrum of existing or repurpose compounds. As the cost of new drug discovery continues to rise, drug repurposing has emerged as a practical approach to identify novel anti-trematode drugs, particularly in resource-limited settings [52,53]. In our pilot screen of 320 compounds, 25 active substances were identified, among which ivermectin demonstrated reproducible efficacy. In conclusion, IVM identified using HTS exhibited significant trematocidal activity against *C. sinensis* in both in vitro and in vivo. Although its efficacy appears limited to the early stage of *C. sinensis* infection, these findings suggest that IVM could be a potential candidate for alternative or combination-based treatment strategies against clonorchiasis.

## 4. Materials and Methods

### 4.1. Chemical Reagents

IVM (Cay355185) and PZQ (Cay20508) were purchased from Cayman Chemical (Ann Arbor, MI, USA) and used to assess in vitro and in vivo anthelmintic effects. For in vitro studies, both compounds were dissolved in dimethyl sulfoxide (DMSO; Sigma-Aldrich, St. Louis, MO, USA). Stock solutions of 100 mM were prepared and stored at −20 °C until use.

### 4.2. Acquisition of C. sinensis Metacercariae and Juvenile Worms

*C. sinensis* metacercariae (CsMC) and newly excysted juvenile worms (CsNEJs) were obtained as previously described [54]. Briefly, *Pseudorasbora parva* infected with *C. sinensis* were homogenized and digested in 1% pepsin-HCl solution at 37 °C for 3 h. The digested material was centrifuged, and supernatants were discarded. Sedimented pellets were repeatedly washed with normal saline, and CsMC were identified microscopically and stored at 4 °C in Locke’s solution. Excystation was induced by incubating CsMC with 0.01% trypsin (Becton Dickinson, Franklin Lakes, NJ, USA), and fresh CsNEJs were prepared immediately prior to each experiment. CsNEJs were used for imaging-based analysis and to assess the in vitro anthelmintic efficacy against excysted metacercariae.

### 4.3. Application of HTS to the Compound Library

A compound library comprising 320 compounds was obtained from commercial sources, including Enamine, Microsource, Prestwick, Tocris, Sigma, and SelleckChem. The library covers bioactive compounds and target-annotated and Food and Drug Administration-approved drugs. All compounds were dissolved in 100% DMSO and stored at −20 °C until use.

### 4.4. Anti-CsNEJs Compound Screening

On the day of screening, compound plates were thawed, centrifuged at 1000 rpm (RT) for 1 min, and 250 nL of each compound was transferred into the assay plate using an automated liquid handling system (Hummingbird, Analytik Jena, Jena, Germany). CsNEJs were obtained by treating CsMC with 0.01% trypsin at 37 °C, followed by washing with Locke’s solution and centrifugation at 1000 rpm for 1 min. Parasites were then seeded at a density of 30 CsNEJs in 25 μL of medium per well onto 384-well μClear plates (Greiner Bio-One, Kremsmünster, Austria) preloaded with compounds. After 24 h of incubation, parasites were fixed with 4% paraformaldehyde and stained with fluorescein diacetate (FDA) + propidium iodide (PI) or calcein AM + DRAQ5. Fluorescence images were acquired using an automated confocal microscope (Opera High-Content Screening System, PerkinElmer, Hamburg, Germany). Nine images per well were captured using a 10× objective lens, and data were analyzed using SimA software (PerkinElmer, Shelton, CT, USA).

### 4.5. Assessing the Larvicidal Effect of IVM Against CsMC and CsNEJs In Vitro

In brief, 100 µL of Locke’s solution containing either 50 CsMC or 50 CsNEJs was dispensed into each well of a 96-well plate. After allowing the parasites to settle, wells were treated with IVM or PZQ at concentrations of 12.5, 25, 50, and 100 µM. A vehicle control containing 1% DMSO in Locke’s solution was also included. Parasite viability was assessed at 24, 48, and 72 h post-treatment using a Leica DMi8 microscope (Leica, Wetzlar, Germany). To evaluate drug effects on CsNEJs motility, juvenile worms were exposed to the same concentrations of IVM and PZQ for 24 h and categorized into four stages: dead, viable but immobile, weakly motile, and highly motile. Motility scoring was used as an additional parameter to assess drug efficacy. Parasite viability and morphological changes were further visualized using fluorescence microscopy.

$$Viability\left(\%\right)=\frac{Live\;worms\;(FDA)}{\left[Live\;worms\left(FDA\right)+Dead\;worms\;(PI)\right]}\times 100$$

### 4.6. IVM Efficacy Against C. sinensis in Rats

All experimental procedures were approved by the Institutional Animal Care and Use Committee (IACUC) of Kyung Hee University (permit ID: KHSASP-24-052), and all efforts were made to minimize animal suffering. A total of 24 five-week-old male rats were obtained from Koatech (Pyeongtake, Republic of Korea). Twenty rats were randomly assigned to an infection group, while the remaining four were assigned to a control group (*n* = 4). Rats in the infection group received 50 CsMC orally via gavage and were further divided into an infection control group (*n* = 4), a PZQ treatment group (*n* = 8), and an IVM treatment group (*n* = 8). To evaluate the trematocidal effect, rats were treated with IVM (30 mg/kg) at either 1 or 4 weeks post-infection (wpi). The PZQ treatment group received 250 mg/kg at the same time points. Control rats received an equivalent volume of 10% DMSO orally. At 4 or 8 wpi, animals were sacrificed, and worms were recovered from the bile ducts. For serum collection, blood samples were collected via retro-orbital plexus puncture, allowed to clot at room temperature for 1 h, centrifuged at 2000 rpm for 10 min, and subsequently stored at −80 °C until use.

### 4.7. C. sinensis-Specific IgG Antibody Response

To evaluate parasite-specific antibody responses, an enzyme-linked immunosorbent assay (ELISA) was performed as described [55,56]. Briefly, 96-well plates were coated with 5 µg/mL of *C. sinensis* excretory/secretory proteins (CsESPs) in 100 µL of carbonate coating buffer (15 mM Na_2_CO_3_, 34.8 mM NaHCO_3_) at 4 °C overnight. The plates were washed three times with phosphate-buffered saline (0.15 M; pH 7.5–7.6) containing 0.05% Tween 20 (PBST). Nonspecific binding sites were blocked with 1% bovine serum albumin (BSA) prepared in PBST at 37 °C for one hour. After additional washes, 100 μL of serum samples, diluted 1:100 in PBST, were added to the plates and incubated at 37 °C for one hour. The plates were then washed again and incubated with 100 μL of goat anti-hamster IgG secondary antibody conjugated with horseradish peroxidase (1:2000 dilution in PBST) at 37 °C for 1 h. The reaction was developed using hydrogen peroxide and orthophenylene diamine (0.04% in phosphate-citrate buffer 0.1 M, pH 5) as the chromogenic substrate. Absorbance values were measured at 450 nm using a microplate reader (Molecular Devices, San Jose, CA, USA).

### 4.8. Evaluation of Liver Function

Liver fibrosis in different rat groups was evaluated using fibrosis-related parameters, including liver function tests. Serum levels of alanine aminotransferase (ALT) and aspartate aminotransferase (AST) were measured using ELISA kits (ab285264; ab263883; Abcam, Cambridge, UK). The results are expressed as units per liter (U/L).

### 4.9. Quantification of Larval Survival and Worm Burden Reduction

The larval survival rate and worm burden reduction were calculated using the following formulas:

Larval survival rate = [number of live larvae/(number of live larvae + number of dead larvae)] × 100%

### 4.10. Statistical Analysis

All samples were processed on an individual basis, and data are expressed as means ± SD. All statistical analyses were performed using GraphPad Prism 6.0 software (San Diego, CA, USA). Statistical significance between the means of groups was determined using a one-way analysis of variance with Bonferroni’s *post hoc* test or Student’s *t*-test. *p* < 0.05 was considered statistically significant, and this was denoted using asterisks (* *p* < 0.05, ** *p* < 0.01, and *** *p* < 0.001).

## 5. Conclusions

In summary, IVM identified using HTS exhibited dose-dependent trematocidal activity against *C. sinensis* metacercariae (CsMC) and newly excysted juveniles (CsNEJs). In rat study, IVM revealed a significant worm burden reduction compared to untreated control, showing an antiparasitic effect against larvae. Although its efficacy appears limited to the early stage of *C. sinensis* infection, these findings suggest that IVM could be a potential candidate for alternative or combination-based treatment strategies against clonorchiasis.

## Data Availability

The original contributions presented in this study are included in the article. Further inquiries can be directed at the corresponding author.

## Abbreviations

The following abbreviations are used in this manuscript:

CsMC	*C. sinensis* metacercariae
CsNEJs	Newly excysted juveniles of *C. sinensis*
IVM	Ivermectin
HTS	High-throughput screening
wpi	Week post-infection
ALT	Alanine aminotransferase
AST	Aspartate aminotransferase
BSA	Bovine serum albumin
CsESPs	*C. sinensis* excretory-secretory proteins
PZQ	Praziquantel
CCA	Cholangiocarcinoma
NTS	Newly transformed schistosomula
